# Broadband THz Molecular Fingerprinting Enabled by Brillouin Zone Folded Quasi‐BICs in Plasmonic Metasurfaces

**DOI:** 10.1002/nap2.70076

**Published:** 2026-04-16

**Authors:** Yihan Xu, Xieyu Chen, Shoujun Zhang, Lu Zhou, Zhen Tian

**Affiliations:** ^1^ Center for Terahertz Waves and College of Precision Instrument and Optoelectronics Engineering, State Key Laboratory of Precision Measuring Technology and Instruments Tianjin University Tianjin China; ^2^ School of Chemistry and Pharmaceutical Engineering Shandong First Medical University & Shandong Academy of Medical Sciences Jinan China

**Keywords:** bound states in the continuum, broadband, fingerprint detection, metasurfaces, sensors, terahertz

## Abstract

Metasurfaces have emerged as a revolutionary platform for high‐performance terahertz (THz) sensing, particularly for biochemical trace detection. However, traditional metasurface sensors are fundamentally constrained by their inherent narrowband response, which restricts their molecular identification to isolated resonance frequencies. In this study, we propose a broadband and high‐sensitivity THz fingerprint metasensor by exploiting the scalability of perturbed Brillouin zone folding bound states in the continuum (BZF‐BIC). The design utilizes a mirror–symmetric, dual‐plasmonic elliptical resonator structure where a *C*_2_ symmetry‐breaking perturbation is introduced to excite high‐*Q* scalable quasi‐BIC (q‐BIC) modes characterized by strong localized field enhancement. Crucially, unlike traditional single‐frequency detection, our strategy establishes a series of high‐*Q* resonant probes across discrete frequency channels, enabling the high‐sensitivity retrieval of molecular absorption fingerprints over a broad THz regime. Numerical simulations demonstrate that the proposed metasensor achieves an average 14‐fold enhancement in the extracted molecular absorption signal compared to bare substrate across 0.3 to 0.8 THz, facilitating the accurate identification of *α*‐lactose, *cis*‐azobenzene (*cis*‐AB), and benzoic acid. Furthermore, the sensor's capability for trace molecule identification and quantitative concentration analysis is experimentally validated using *cis*‐AB. This work offers a robust and versatile avenue for highly sensitive, broadband on‐chip THz spectroscopic analysis systems.

## Graphical abstract

A broadband terahertz fingerprint metasensor is proposed based on Brillouin zone folding‐driven quasi‐bound states in the continuum. By establishing a distributed array of high‐*Q* resonant probes, this sensor achieves a 14‐fold sensitivity enhancement and enables high‐fidelity retrieval of molecular fingerprints across 0.3–0.8 THz, offering a versatile platform for trace biochemical detection.

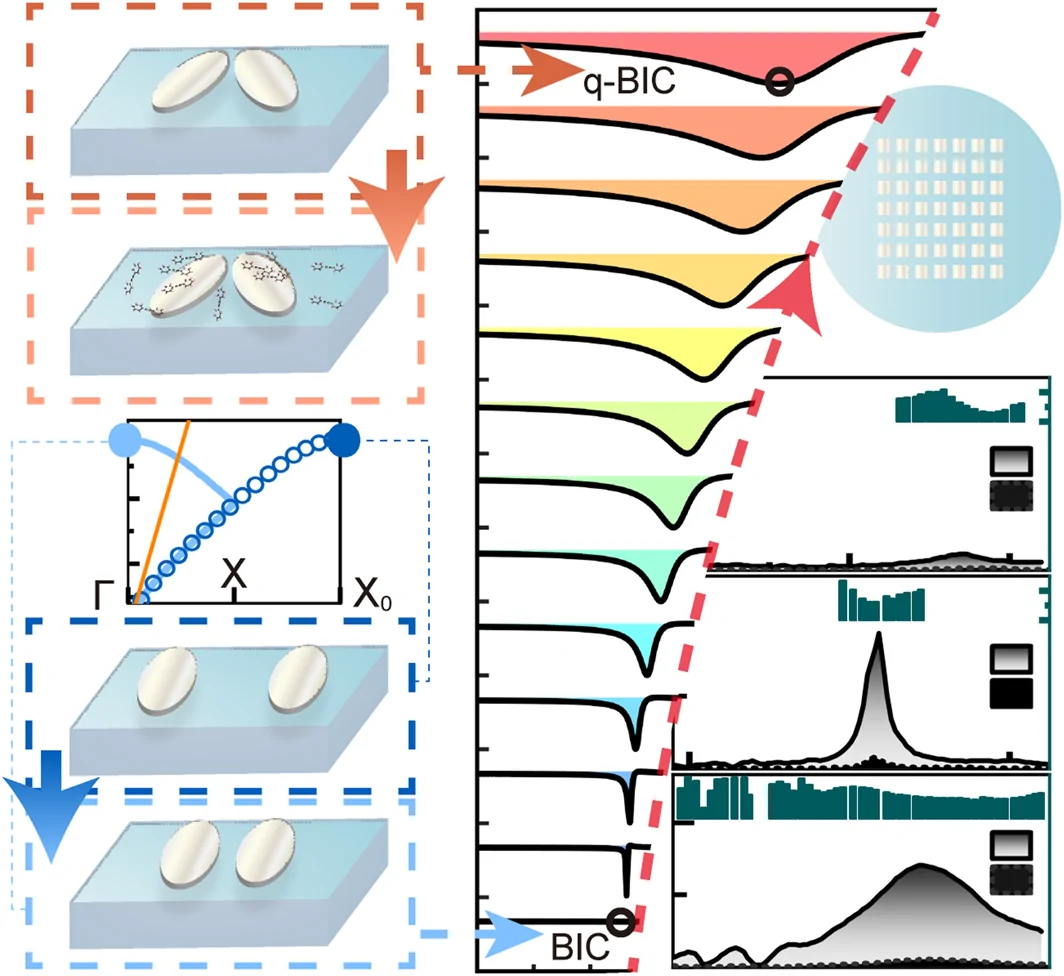

## 1 Introduction

Terahertz (THz) spectroscopy has garnered significant attention for sensing applications owing to its unique properties, including label‐free detection, nondestructive analysis, and nonionizing radiation [1, 2, 3]. Nevertheless, the sensitivity of THz sensing is often constrained by weak light–matter interactions arising from the substantial mismatch between sub‐millimeter THz wavelengths and the nanometric dimensions of trace analytes [1]. Metasurfaces, characterized by sub‐wavelength artificial microstructures, offer an effective avenue to address this challenge by enabling strong electromagnetic field localization and enhancement [4, 5], as well as versatile wavefront and polarization manipulation [6, 7]. The presence of an analyte will perturb the near‐field dielectric environment of the metasurface, inducing detectable variations in its resonant characteristics [8, 9]. Consequently, trace biochemical substances can be identified by monitoring spectral deviations [10, 11, 12, 13], specifically shifts in resonant frequency [14, 15, 16] or amplitude modulation [17, 18, 19].

To improve THz sensing performance, various metasurface structures supporting high‐quality (*Q*) resonances and strong field enhancement have been proposed in recent years, including those utilizing Fano modes [14, 20], toroidal modes [21, 22, 23], and electromagnetically induced transparency [24, 25]. However, these resonant modes typically suffer from limited *Q* factors due to inherent radiative and ohmic losses [26], which fundamentally hinders the ultrasensitive detection of low‐concentration biomolecules. Bound states in the continuum (BICs) emerge as a breakthrough mechanism, theoretically supporting infinite *Q* factors by completely suppressing radiative leakage [27, 28]. By introducing symmetry‐breaking perturbations, these ideal BICs are transformed into quasi‐bound states in the continuum (q‐BICs) [29, 30], enabling the experimental realization of high‐*Q* resonances with intense near‐field enhancement. These unique properties establish q‐BIC metasurfaces as ideal candidates for trace molecular fingerprint retrieval.

However, a major constraint persists in target‐specific sensing applications: optimal sensitivity relies heavily on the precise spectral overlap between the analyte's molecular absorption peak and the metasurface sensor's resonance peak [31]. Consequently, existing metasurface sensors are often carefully designed for specific target resonance frequencies [32, 33, 34]. To overcome this inherent narrowband limitation, several broadband sensing strategies have been proposed. Multi‐resonant metamaterials integrate multiple resonators within a single unit cell to provide several discrete detection channels [32, 35]; however, they suffer from mutual near‐field coupling and typically fail to offer dense, continuous sampling across a wide spectral band [36]. Chirped metasurfaces broaden the spectral response by continuously varying structural parameters across the array, but the collective response of such nonuniform structures inevitably broadens the resonance linewidth, thereby degrading the overall *Q* factor and near‐field enhancement [37, 38]. Recently, spatially multiplexed sensors [39], particularly hyperspectral dielectric metasurfaces [40], have emerged as powerful tools for broadband fingerprinting. Nevertheless, translating these advanced concepts into the terahertz regime encounters practical bottlenecks. Specifically, THz all‐dielectric designs require high‐aspect‐ratio microstructures [41, 42] that severely complicate uniform analyte coating, whereas conventional spatially multiplexed plasmonic sensors often struggle to maintain the ultra‐high *Q* factors required for high‐resolution sampling [39]. Therefore, achieving high‐sensitivity broadband THz fingerprinting requires a novel resonance mechanism that can robustly maintain ultra‐high *Q* factors during massive dimensional scaling, which remains a significant challenge.

In this work, we propose and demonstrate a broadband, high‐sensitivity THz metasensor by leveraging the scalable high‐*Q* characteristics of q‐BIC modes within a BZF‐BIC configuration. The metasurface, consisting of perturbed mirror‐symmetric dual‐plasmonic elliptical resonators, is designed to excite at‐Γ BZF‐BICs. We analyze the nonradiative nature of the BZF‐BIC by investigating the radiating and topological properties of the eigenmodes. By introducing structural perturbations, this *C*_2_ symmetry‐protected state is transformed into a radiative q‐BIC mode characterized by a scalable high *Q* factor and strong near‐field enhancement. Departing from conventional single‐frequency detection, our strategy establishes a “distributed” sensing platform comprising 49 high‐*Q* resonant probes that uniformly span the broad 0.3–0.8 THz range. When coated with an analyte, the frequency‐specific molecular absorption modulates the response of the corresponding resonant probes, allowing for the high‐fidelity reconstruction of the complete THz absorption fingerprint. We numerically demonstrate this capability by retrieving the fingerprint spectra of *α*‐lactose, *cis*‐azobenzene (*cis*‐AB), and benzoic acid. Furthermore, the sensor's performance is experimentally validated through device fabrication and the successful capture of the broadband absorption spectrum of *cis*‐AB. These results demonstrate that the proposed metasensor is a powerful tool for the sensitive and broadband retrieval of molecular fingerprints from trace substances.

## 2 Principle and Design

### 2.1 Physical Mechanism of BZF‐BIC and the Manipulation of q‐BIC Modes

An ideal BIC is a perfectly confined, nonradiative eigenmode that coexists with the continuous spectrum of radiating waves, theoretically possessing an infinite *Q* factor [27]. Because ideal BICs are completely decoupled from free space, they are typically transformed into observable q‐BICs by introducing structural perturbations that break the spatial symmetry, yielding resonances with finite but ultra‐high *Q* factors and intense near‐field enhancement [30]. Among various mechanisms to construct such states, the BZF‐BIC represents a specific class of symmetry‐protected BICs formed by engineering the lattice periodicity to fold nonradiative guided modes from the Brillouin zone edge to the Γ‐point [43]. Compared to conventional guided‐mode resonances, BZF‐BICs provide a broader tuning range and additional degrees of freedom to manipulate the *Q* factor. Furthermore, unlike standard symmetry‐protected BICs, the *Q* factor of a BZF‐BIC is governed by both the engineered perturbation parameter and the wavevector. This dual dependence endows the resonance with superior angular robustness and enhanced resilience against structural disorder and fabrication imperfections [43, 44]. Leveraging these fundamental principles, we designed a metasurface platform to physically realize and manipulate these states for sensing applications.

The proposed metasurface unit cell consists of an aluminum elliptical resonator on a silicon substrate. As shown in Figure 1a, two adjacent metasurface unit cells are schematically illustrated. Numerical simulations were conducted using the commercial software COMSOL Multiphysics to study the eigenmodes. A guided mode (GM) below the light cone (orange line) can be observed on the band structure along Γ‐*X*_0_, depicted as blue hollow circles in Figure 1c. By introducing a periodic gap perturbation Δ*d* between adjacent elliptical resonators, the periodicity of the metasurface in the *x* direction can be doubled, as shown in Figure 1b. This perturbation reduces the first Brillouin zone dimension by half, as illustrated in the inset of Figure 1c, folding the *X*_0_‐point onto the Γ‐point. According to the selection rules for engineering symmetry‐protected BICs by folding high symmetry modes to Γ‐points [45], the original GM enters the radiation continuum above the light line while remaining nonradiative, therefore forming a BZF‐BIC. As a schematic indication, the perturbed band structure with perturbation factor *α*_1_ = 0.006 (defined as *α*_1_ = Δ*d*/*d*_0_, corresponds to a physical gap perturbation of Δ*d* = 0.4 μm) is plotted as a magenta solid line in Figure 1c.

**FIGURE 1:**
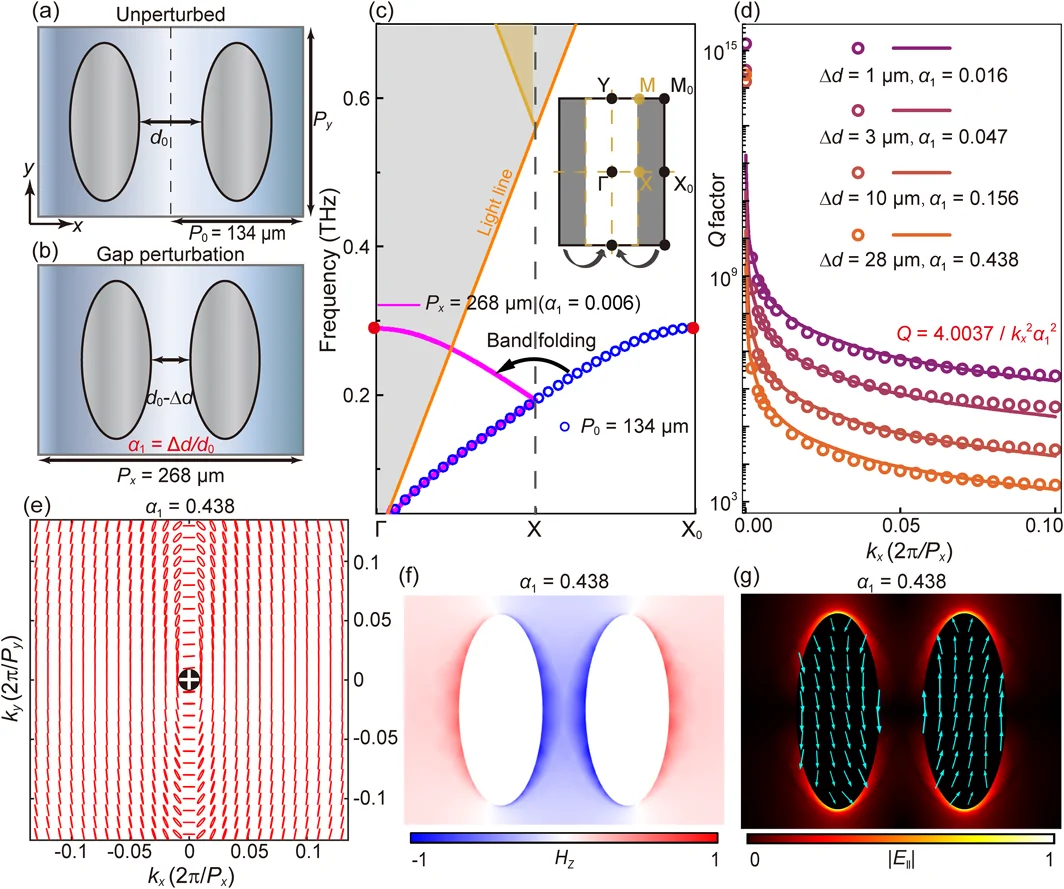
Physical mechanism of BZF‐BIC. (a) Lattice geometry without perturbation, with parameters *P*_0_ = 134 μm, *P*_*y*_ = 192 μm, and *d*_0_ = 64 μm. (b) Lattice geometry with introduced gap perturbation (Δ*d*), resulting in a doubled period *P*_*x*_ = 268 μm. (c) Calculated band structures along Γ‐*X*_0_ for the unperturbed (blue hollow circles) and gap‐perturbed (magenta solid line) lattices with perturbation factor *α*_1_ = 0.006. The black arrow indicates the folding of the *X*_0_‐point to the Γ‐point, forming the BZF‐BIC. The inset shows that the first Brillouin zone is halved upon perturbation. The shaded gray area represents the light cone in air. (d) Simulated *Q* factors of the folded band with *kx* varying from 0 to 0.1 under different perturbation factors *α*_1_. The solid lines are inverse quadratic fits to the simulated data ($Q\;\propto \;1/k_{x}^{2}\alpha_{1}^{2}$). (e) Simulated far‐field polarization map for the BZF‐BIC for a gap perturbed lattice (*α*_1_ = 0.438), carrying a +1 topological charge at the center of Brillouin zone. Calculated (f) out‐of‐plane magnetic field profile *H*_*z*_ and (g) in‐plane electric field profile $\left|E_{∥}\right|$ overlaid with surface current distributions (cyan arrows) of the eigenmode at Γ‐point for the perturbed lattice with *α*_1_ = 0.438.

The radiative property of the BZF‐BIC mode was subsequently investigated. As illustrated in Figure 1d, the *Q* factor of BZF‐BIC approaches infinity at Γ‐point and decreases as the wavevector deviates from the Γ‐point in the momentum space. Moreover, the *Q* factor exhibits significant modulation under different perturbation factors *α*_1_. Further study reveals that the *Q* factor follows an inverse quadratic law to both wavevector and perturbation parameter and can be fitted to the function $Q=4.0037/k_{x}^{2}\alpha_{1}^{2}$. This radiative property is consistent with the BZF‐BIC modes reported in previous works [43, 46], providing an additional degree of freedom for engineering the *Q* factor. Furthermore, the far‐field polarization map near the Γ‐point of BZF‐BIC is plotted in Figure 1e. A significant polarization singularity carrying a topological charge of *q* = +1 can be observed at the Γ‐point, fully demonstrating the topologically protected property of proposed BZE‐BIC mode. Here, the topological charge is defined as $q=\left(1/2\pi \right)\int_{C}\nabla k\Phi \left(k\right)dk$, where Φ(k) is the angle between the polarization major axis and *x*‐axis, and *C* is a simple closed counter‐clockwise path around the BIC in momentum space [47]. The nonradiative nature of the BZF‐BIC can be understood through its field distributions, as illustrated in Figure 1f, which exhibit even symmetry under *C*_2_ rotation. The symmetry incompatibility between this even–parity mode and odd–parity radiation channel fundamentally prevents energy leakage [43]. Also, the antiparallel surface currents of BIC mode (cyan arrows in Figure 1g) induce a dominant longitudinal magnetic dipole (MD) moment perpendicular to the metasurface. Since this vertical MD mode cannot couple to transverse free‐space waves, radiative leakage is strictly forbidden, confirming the symmetry‐protected BIC [46].

The proposed BZF‐BIC is an in‐plane inversion (*C*_2_) symmetry‐protected dark mode that cannot be directly observed in the far‐field spectrum due to its complete suppression of radiative loss. To activate this mode for sensing applications, we introduce a controlled structural perturbation to break its symmetry, thereby transforming it into an observable q‐BIC mode with a finite radiation rate [15]. Here, we employ a “geometric rotation” strategy for symmetry breaking, which involves symmetrically rotating the pair of elliptical resonators within a unit cell in opposite directions by an angle *θ* with respect to the *y*‐axis to break the *C*_2_ symmetry.

To thoroughly understand this tuning mechanism, we systematically investigated the influence of the rotation angle *θ* on the modal properties, as shown in Figure 2. In this analysis, the base metasurface geometry is utilized, denoted by a dimensional scaling factor *S* = 1. The gap perturbation is fixed at Δ*d* = 12 μm (*α*_1_ = 0.188). This specific value was chosen to provide a sufficiently strong band‐folding perturbation while maintaining an adequate physical gap between the resonators, ensuring that they do not physically overlap or pose fabrication challenges when rotated to large angles (e.g., *θ* = 24°). It is worth noting that this is a representative value; selecting other appropriate gap perturbations would not alter the general physical phenomena and conclusions presented in Figure 2. The transmission spectra in Figure 2a clearly illustrate the evolution of the modal spectral characteristics with the rotation angle *θ*. For *θ* = 0°, the ideal BIC is invisible due to complete radiation suppression. For nonzero *θ*, a sharp resonant dip induced by the q‐BIC mode emerges. With increasing *θ*, the resonance dip shifts to shorter wavelengths and then gradually broadens. This spectral evolution is governed by the modal physical properties under different *θ*. As shown in the complex eigenfrequency analysis in Figure 2e, the imaginary part of the eigenfrequency (represented by the error bar width), which quantifies the radiative loss, monotonically increases with rotation angle *θ*. The increase in radiative loss directly leads to two observable consequences: First, the *Q* factor of mode exhibits a strict inverse quadratic relationship with the asymmetry parameter $\alpha_{2}=\sin\left(\theta \right)$, fitted by $Q=4.1474/\alpha_{2}^{2}$ (Figure 2f), which is consistent with reported work [30]. Second, the localized field enhancement factor around the resonator undergoes a significant reduction, as visualized in Figure 2b–2d. These results reveal the physical mechanism for precisely controlling the radiative properties and thus the sensing performance of the q‐BIC mode through asymmetry parameter.

**FIGURE 2:**
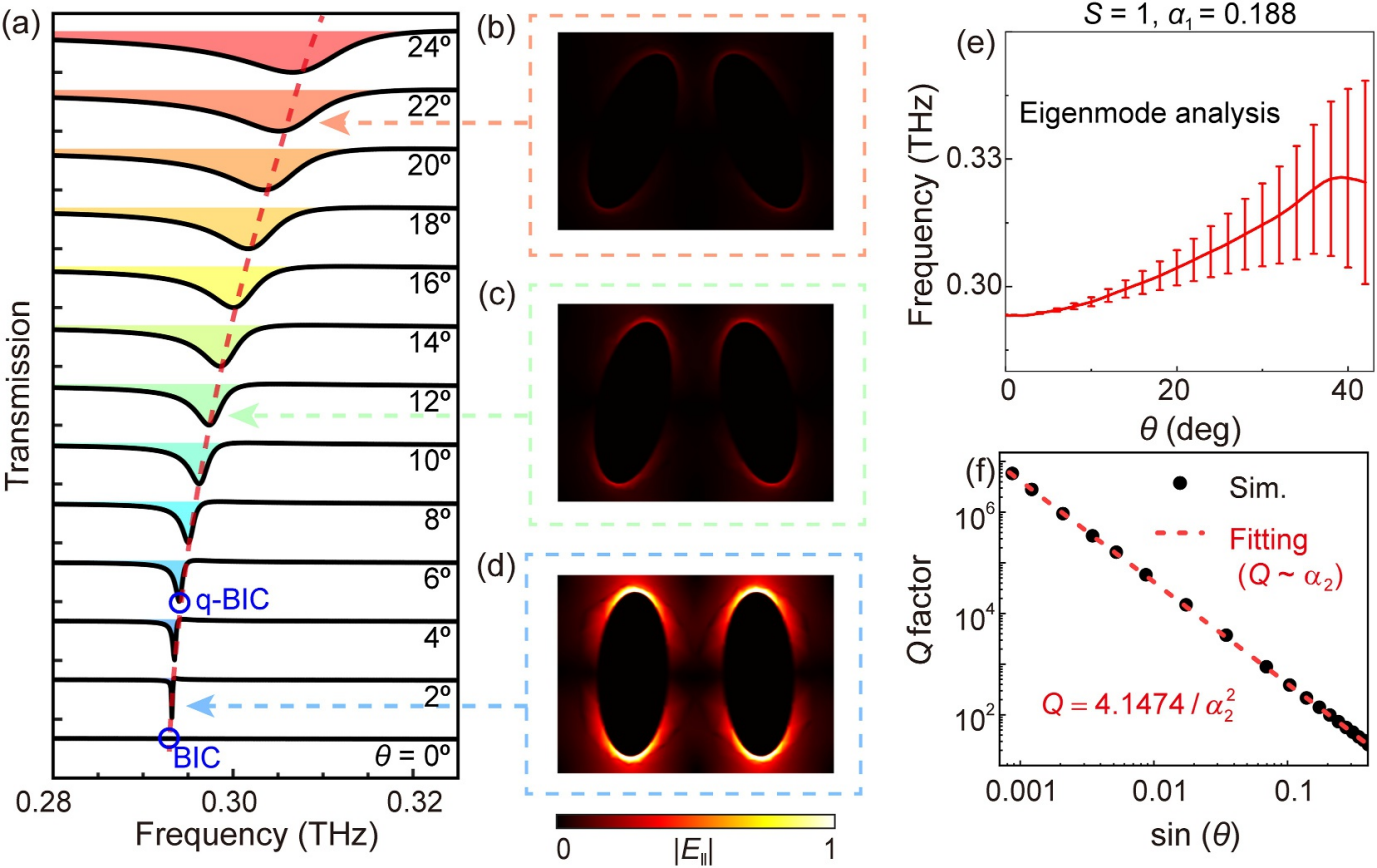
Control of the symmetry‐broken q‐BIC. (a) Simulated transmission spectra as a function of the rotation angle *θ*. The spectra are vertically offset for clarity. A sharp resonant dip emerges and broadens as *θ* increases from 0° to 24°. (b–d) In‐plane electric field distributions $\left(|E_{∥}|\right)$ for different rotation angles. (e) Resonant frequency of the q‐BIC versus rotation angle *θ*. The error bars represent the modal inverse radiation lifetime (the imaginary part of the eigenfrequency). (f) Radiative *Q* factor of the q‐BIC as a function of the in‐plane asymmetry parameter $\alpha_{2}=\sin\left(\theta \right)$. The red dashed line is a fit to the simulation results data (black dots), following the inverse quadratic relationship $Q\;\propto \;1/\alpha_{2}^{2}$.

### 2.2 Design and Simulation of Broadband Sensing Platform

Based on the tuning mechanism discussed above, and by balancing the *Q* factor with experimental feasibility, the structure with *θ* = 24° was selected as the fundamental building block for the broadband sensing platform. The core idea is to realize an array of high‐*Q* resonant dips that act as “frequency sampling probes” uniformly distributed across the fingerprint absorption band of target analytes. To this end, we designed a 7 × 7 metasurface array (Figure 3a), where the geometric dimensions of each array are precisely controlled by a scaling factor *S*. As shown in the Figure 3d, by varying *S* from 0.39 to 1, we successfully obtained 49 sharp resonances with a uniform frequency spacing of 10 GHz, covering the broad spectral range of 0.3–0.8 THz. The approximately inverse relationship between the resonant frequency *f*_0_ and the scaling factor *S* is further demonstrated in Figure 3e. These resonant dips collectively form a high‐resolution sensing baseline, providing a solid foundation for the accurate retrieval of analyte absorption fingerprints.

**FIGURE 3:**
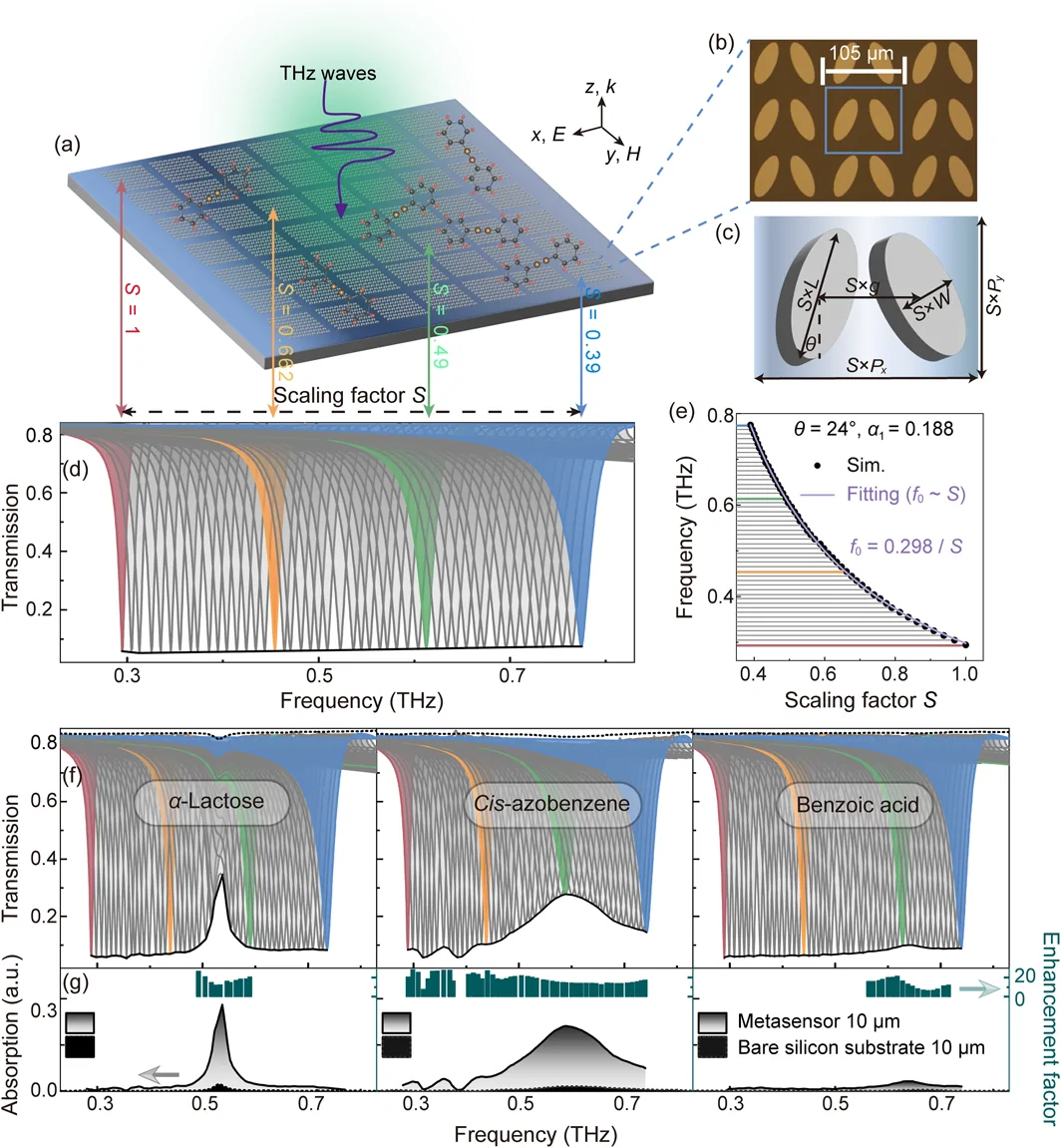
Design and simulation results of the q‐BIC‐based sensor platform. (a) Schematic of the broadband THz molecular fingerprint sensor, featuring a metasurface composed of a high‐*Q* resonator array with resonances tuned by a scaling factor *S* to cover the target molecular fingerprint range. (b) Optical image of the fabricated metasurface. (c) Unit cell dimensions. (d) Simulated transmission spectra for different scaling parameter values *S*, showing 49 resonant frequencies spanning from 0.3 to 0.8 THz. (e) Inverse relationship between the resonant frequency *f*_0_ and the scaling factor *S*. The black dots and purple line represent the simulation and fitting results respectively. The horizontal grid lines indicate the uniform spacing of the resonant frequencies. (f) Simulated transmission spectra of the metasurface coated with *α*‐lactose, *cis*‐AB, and benzoic acid. The envelope of the resonant amplitudes (solid black lines) reproduces the absorption peak of the analytes, whereas the dotted lines represent the transmission through the analytes on a bare silicon substrate. (g) Top panels show calculated enhancement factor of the metasurface sensor compared to bare silicon substrate. Bottom panels illustrate retrieved absorption spectra for the three analytes using the metasurface sensor (solid bars) and bare silicon substrate (dotted lines).

For the above design, the spectral response of the metasurface unit cell was simulated and carefully optimized using the commercial software CST Microwave Studio. In the simulations, Floquet periodic conditions were set in the *x* and *y* directions, whereas the open boundary condition was applied in the *z* direction. The metasurface unit cell was excited by an *x*‐polarized THz wave propagating along the *z* direction. As illustrated in Figure 3c, the geometric parameters of the basic unit cell (*S* = 1) were set as follows: periodicities *P*_*x*_ = 268 μm and *P*_*y*_ = 192 μm, resonator thickness *d* = 200 nm, minor axis *W* = 70 μm, major axis *L* = 160 μm, center‐to‐center distance between resonators *g* = 122 μm, and orientation angle *θ* of 24°. Notably, *θ* = 24° was selected as an optimal balance, providing a *Q* factor high enough for sensitive detection while ensuring the resonances were experimentally robust and reliably measurable. For the materials, the resonators were modeled as aluminum with a conductivity of 3.56 × 10^7^ S m^−1^, and the substrate was silicon with a dielectric constant of *ε* = 11.7. Detailed justifications for this material choice and an analysis confirming the negligible impact of the native aluminum oxide layer are provided in S1 of the Supporting Information S1. The complex refractive indices of the three analytes used in the simulation were experimentally obtained by photoconductive‐antenna‐based THz time‐domain spectroscopy (THz‐TDS) for accuracy [48].

The principle of designed metasurface sensor relies on the coupling between the characteristic molecular vibrations of the analyte and the highly localized THz field around the resonators [40]. The interaction selectively modulates the intensity and linewidth of the corresponding resonant dips, thereby imprinting a distinguishable “fingerprint” onto the transmission spectrum. The performance of proposed sensor was first validated by numerical simulations using *α*‐lactose, *cis*‐AB, and benzoic acid as analytes. The simulated transmission spectra for the metasurface coated with a 10 μm layer of three analytes are shown in Figure 3f. It can be observed that the envelope formed by the resonance dips for the three analytes effectively traces their distinct absorption characteristics, enabling the high‐fidelity retrieval of their fingerprint spectra, as illustrated in the bottom panels of Figure 3g. For comparison, we simulated the three analytes directly coated on a bare silicon substrate. The resulting transmission spectra (dotted lines in Figure 3f) and extracted fingerprint spectra (dotted lines in Figure 3g) remain quite flat and exhibits weak absorption features. Further quantitative calculations show that the peak amplitude of the extracted molecular absorption signal for the trace analytes are enhanced by the average factor of over 14 times using the metasurface sensor (calculated as the ratio of the absorption peak amplitude on the metasensor to that on the bare silicon substrate for the same analyte thickness). These simulation results fully validate the high‐sensitivity and broadband molecular fingerprint retrieval capability of proposed sensor platform.

## 3 Results and Discussion

### 3.1 Sample Preparation and Measurement Methods

To experimentally validate the sensing performance of proposed platform, the metasurface sensor was fabricated using standard photolithography and wet etching techniques. First, a 200‐nm‐thick aluminum layer was deposited onto a 2‐mm‐thick high‐resistivity silicon wafer via thermal evaporation. Subsequently, a photoresist layer was patterned using UV lithography, and the aluminum was selectively etched to form the desired metasurface structure. A step‐by‐step fabrication flowchart and detailed procedures are provided in Section S2 of the Supporting Information S1. Figure 3b presents an optical image of the fabricated metasurface, demonstrating excellent pattern fidelity consistent with the design.

For the sensing experiments, *cis*‐AB was selected as the primary analyte because its broad and strong absorption profile yields a high signal‐to‐noise ratio, making it optimal for validating the broadband envelope reconstruction mechanism in practical THz‐TDS measurements. Furthermore, the metasensor is highly reusable and can be regenerated for subsequent tests via prompt solvent washing (see Supporting Information S1: S3 for details). *Trans*‐azobenzene (*trans*‐AB) was purchased from J&K Scientific Co. Ltd. and partially isomerized to *cis*‐AB under UV irradiation. The *cis*‐AB was then purified via column chromatography and diluted with acetone to various concentrations [49]. For spectral measurements, a 2 mL solution of *cis*‐AB was applied to cover the sensor surface, followed by natural evaporation for 2 min to remove the solvent. The thickness of the analyte layer was controlled by the concentration of the drop‐cast solution and was estimated based on the mass conservation principle, using the solution volume and the bulk density of *cis*‐AB (∼1.09 g cm^−3^). For verification, the thickness was also measured using a micrometer screw with a precision of 1 μm by averaging measurements from multiple points across the substrate surface, which shows good consistency with the estimated values.

The performance of the metasurface sensor was characterized using an all‐fiber THz‐TDS system (Advanced Photonics Inc.) with a spectral range of 0.2–1.2 THz. During the measurements, the effective THz beam spot size was strictly defined by a 4‐mm‐diameter aperture on the sample holder. To measure the 49 distinct arrays, the metasurface chip was manually translated to sequentially align each specific array with the aperture, ensuring that the THz beam only illuminated one array at a time without crosstalk. All measurements were conducted at room temperature in a dry atmosphere to minimize water vapor absorption. The amplitude transmission coefficient [19, 48], *t*, was calculated using the equation $t=\left|E_{\text{sam}}\left(f\right)\right|/\left|E_{\text{ref}}\left(f\right)\right|$, where $E_{\text{ref}}\left(f\right)$ and $E_{\text{sam}}\left(f\right)$ represent the electric field amplitude transmitted through free space (air) and the sample, respectively. The transmission spectra were acquired for both the metasurface sensor and the control silicon wafer, before and after analyte deposition.

### 3.2 Experimental Demonstration

Next, we experimentally validated the broadband fingerprint spectrum retrieval capability of the metasurface sensor. The measured transmission spectra of the metasurface before and after being coated with 25 mg mL^−1^ of *cis*‐AB, are presented in Figure 4a,b, respectively. These results demonstrate excellent agreement with the simulations shown in Figure 3d,f (middle panel) in both the dense resonant dip profile of the bare metasurface and the overall elevation of the transmission envelope after analyte deposition. Moreover, Figure 4c shows the measured inverse relationship between the resonant frequency *f*_0_ and the size scaling factor *S* for the fabricated device, which also aligns well with the simulation design in Figure 3e. These experimental results confirm the high quality of the device fabrication and the robustness of the design against potential fabrication tolerances. Furthermore, the absorption fingerprint spectrum was obtained by subtracting the baseline envelope of the bare metasurface (*t*_0_) from that of the sensor coated with sample (*t*_s_), as shown by the solid line in Figure 4d. Specifically, this continuous envelope was reconstructed by applying a standard cubic spline interpolation to the 49 discrete subtracted signal points. A detailed quantitative evaluation of the reconstruction error is provided in S4 of the Supporting Information S1. The resulting positive signal (*t*_s_ − *t*_0_) originates from the analyte‐induced damping of the high‐*Q* resonances and clearly reproduces the absorption features of *cis*‐AB. In contrast, for the conventional sensing configuration, the absorption spectrum was obtained by subtracting the transmission of the analyte‐coated silicon wafer (*t*_s_si_) from that of the bare silicon wafer (*t*_0_si_). However, as depicted by the dashed line in Figure 4d, no obvious absorption peak is detectable by conventional sensing method. These measurement results highlight the significant sensitivity enhancement effect and broadband fingerprint spectrum retrieval capability of the proposed metasurface sensor in the THz regime. Moreover, the design can be further extended to a broader spectral region by tuning its geometrical parameters.

**FIGURE 4:**
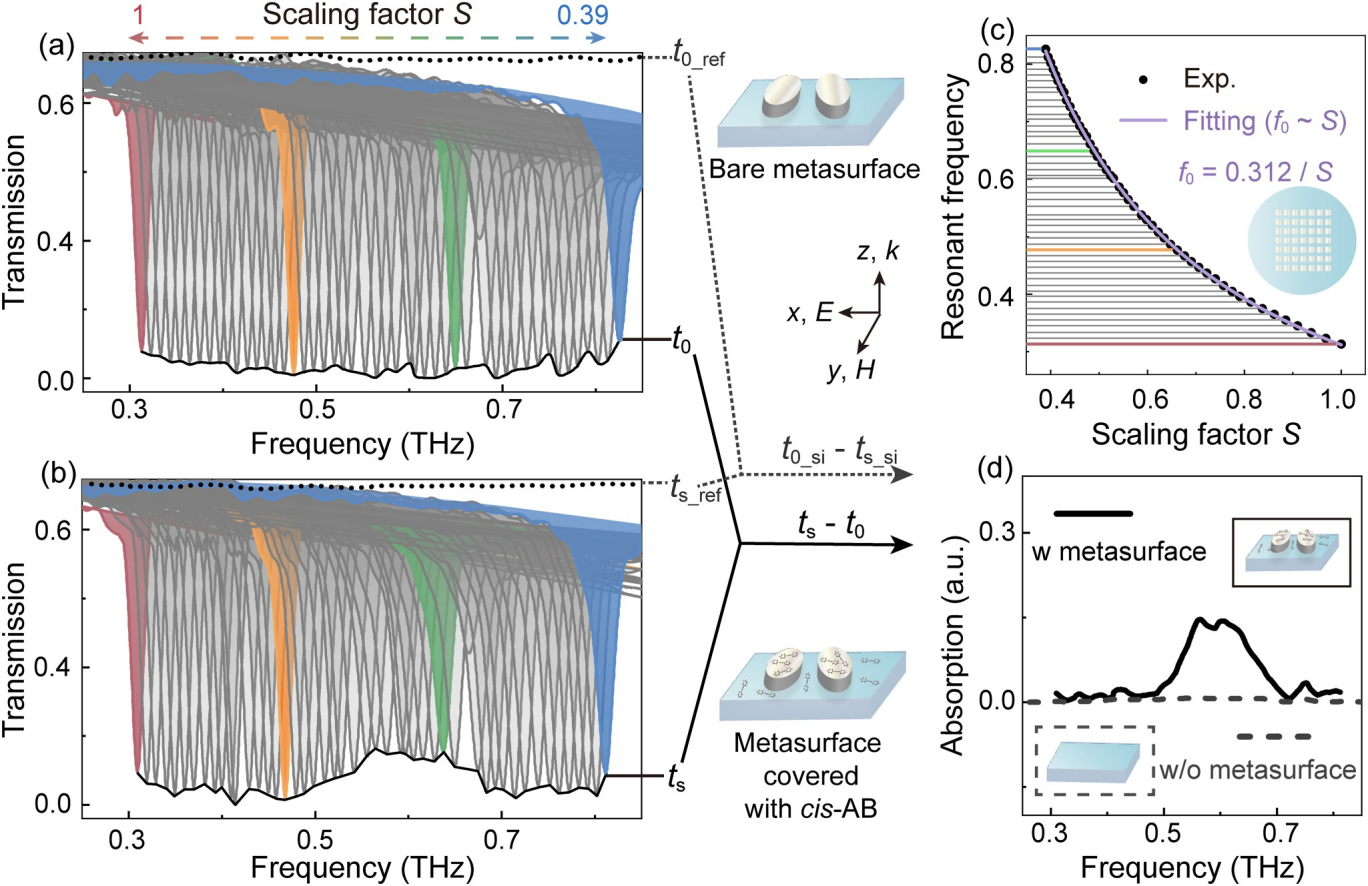
Experimental demonstration of analyte fingerprint retrieval. Measured transmission spectra of (a) bare metasurface sensor and (b) metasurface sensor coated with 25 mg mL^−1^ *cis*‐AB. The weakened resonance of the 35th curve is attributed to a localized fabrication defect. (c) Measured inverse relationship between resonant frequency *f*_0_ and size scaling factor *S* for the fabricated device. The black dots and purple line represent the experimental and fitting results respectively. The horizontal grid lines indicate the uniform spacing of the resonant frequencies. (d) Retrieved fingerprint spectra of *cis*‐AB by the metasurface sensor (solid line) and the bare silicon substrate (dashed line).

Beyond specific molecular recognition, our device also demonstrates the capability for quantitative analysis. Figure 5a illustrates the numerically retrieved fingerprint spectra of *cis*‐AB with varying thickness using the metasurface sensor. For different thicknesses, distinct absorption intensities can be observed in the 0.5–0.7 THz range. Moreover, Figure 5b presents the experimentally retrieved fingerprint spectra as the concentration of *cis*‐AB on the metasurface increases from 10 to 25 mg mL^−1^. For higher concentrations of analyte, the absorption of THz increases and thus the amplitude of retrieved absorption spectra, agreeing well with the overall trend of the simulation. However, small discrepancies between the experimental and simulated spectra can be observed, which are primarily attributed to the nonideal uniformity of the drop‐casted analyte film, as well as the inherent noise floor and long‐term stability fluctuations of the THz‐TDS system during the sequential measurement of the 49 arrays. To quantitatively examine the relationship between analyte thickness and THz absorption, the peak amplitude of the simulated fingerprint spectra was extracted and plotted as a function of *cis*‐AB thickness, as shown by black dots in Figure 5c. The simulated data was fitted using an exponential saturation model, which reflects the decay of the evanescent field extending from the metasurface [50]. The fitting equation is given as $\Delta A=\Delta A_{\max}\left(1-e^{-h/d_{\text{decay}}}\right)$, where Δ*A*_max_ represents the saturation amplitude and $d_{\text{decay}}$ corresponds to the decay length of the local field. The fit shows excellent agreement (*R* ^2^ ≈ 0.98) with the simulation data. The saturation behavior is consistent with the finite penetration depth of the fringing fields confined around the resonators [19]. The results indicate that for analyte thicknesses below 20 μm, the extracted absorption peak increases sharply with increasing thickness, demonstrating the sensor's high sensitivity to thin analyte layers. However, for analyte thickness exceeding 20 μm, the increase in extracted absorption peak saturates due to the mismatch between the analyte thickness and the strong electric field region [51, 52], as shown in the gray‐shaded area. This makes the metasurface sensor particularly suitable for trace detection. Moreover, the peak amplitude of the measured absorption spectra was also extracted and added into Figure 5c, as illustrated by green triangles, which exhibits excellent qualitative agreement with the simulation. These results conclusively demonstrate the metasurface sensor's capability for quantitative detection of trace analytes.

**FIGURE 5:**
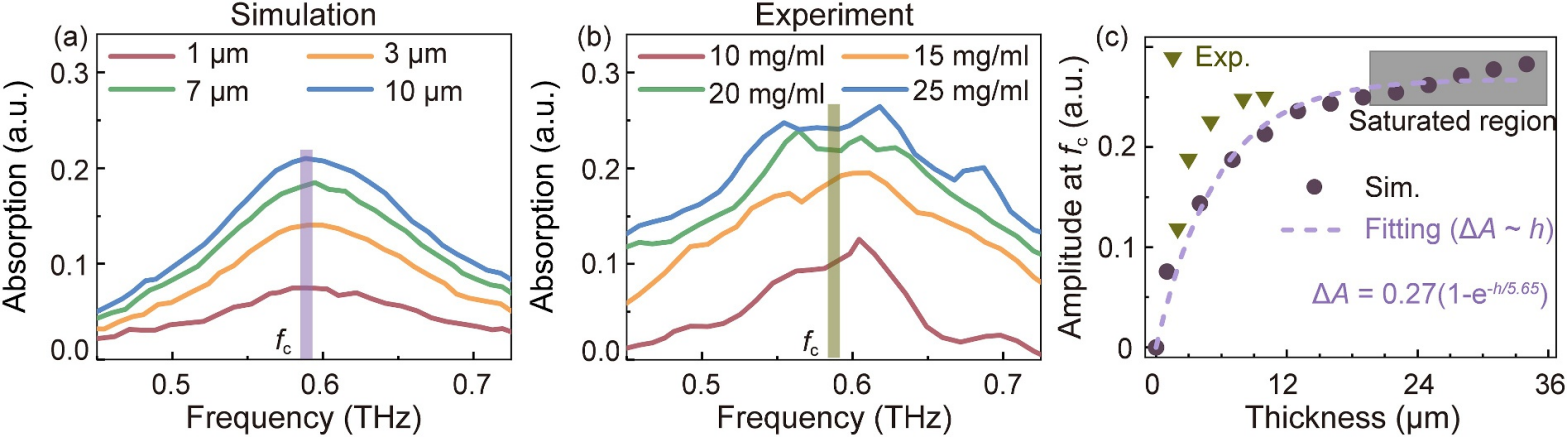
Quantitative detection of the analyte. (a) Numerically retrieved and (b) experimentally retrieved fingerprint spectra of *cis*‐AB with different thicknesses and concentrations, using the metasurface sensor. (c) Simulated (black dots) and measured (green triangles) amplitudes of the retrieved fingerprint spectra at the central absorption frequency (*f*_c_), plotted as a function of *cis*‐AB thickness. The dashed purple line is the fit to the simulation data using the exponential saturation model ($\Delta A=0.27\left(1-e^{-h/5.65}\right)$). The thicknesses corresponding to the four experimental concentrations (10, 15, 20, and 25 mg mL^−1^) were measured to be 2, 3, 5, and 8 μm, respectively.

### 3.3 Comparison and Advantages of the Proposed Metasensor

To further highlight the novelty and practical superiority of the proposed BZF‐BIC metasensor, we compare our platform with recently reported spatially multiplexed (pixelated) metasensors, as summarized in Table 1. Pioneering pixelated all‐dielectric metasurfaces (e.g., Tittl et al. [40]) have shown great success in the mid‐IR regime. However, scaling all‐dielectric designs to the THz regime requires high‐aspect‐ratio resonators (often tens of micrometers thick). Such highly uneven surface topographies can lead to inhomogeneous analyte films during liquid drop‐casting and make the sensor challenging to clean for reuse. In contrast, our 200‐nm‐thick aluminum metasurface is quasi‐planar, ensuring uniform film formation and facilitating straightforward solvent washing. Furthermore, unlike dielectric modes where energy is largely confined inside the pillars, our metallic BZF‐BIC strongly localizes the electric field into the gaps, creating intense surface hotspots that maximize the interaction volume with trace analytes. Compared to pixelated metallic sensors based on conventional resonances like toroidal dipoles [39], our platform leverages the unique physics of BZF‐BICs. The BZF‐BIC provides a theoretically infinite *Q* factor deterministically governed by both the engineered perturbation parameter and the wavevector. This dual dependence endows the resonance with superior robustness against structural disorder, enabling reliable unit‐cell scaling across a broad spectrum without resonance degradation. Additionally, our use of natively adherent aluminum eliminates the need for lossy adhesion layers (e.g., Ti or Cr) typically required for gold‐based sensors, further preserving the high‐*Q* performance.

**TABLE 1:** Comparison of the proposed metasensor with recently reported spatially multiplexed (pixelated) metasensors.

Reference	Operating band	Material platform	Resonance mechanism	Multiplexing strategy	Performance (EF/LOD)	Analyte and validation
Tittl et al. [40]	Mid‐IR (1350–1750 cm^−1^)	All‐dielectric (a‐Si:H)	Quasi‐BIC/Mie	Pixelated array (100 pixels)	LOD: 2130 [molecules/μm^2^]	Protein A/G (Exp.)
Han et al. [39]	THz (1.5–2.3 THz)	Au (with 10 nm Ti)	Toroidal dipole	Pixelated array (25 pixels)	EF: 10.4×	Glucose (Exp.)
Lyu et al. [53]	THz (0.95–2.0 THz)	Au (on quartz)	AIT	Pixelated array (36 pixels)	EF: 7.3×	Chiral carnitine (Exp.)
Sun et al. [54]	THz (1.2–2.7 THz)	Au + graphene	Toroidal dipole	Pixelated array (6 pixels)	EF: ∼5×	Glucose (Sim.)
Hadi Badri et al. [55]	Mid‐IR (1350–1750 cm^−1^)	All‐dielectric (a‐Si:H)	Fano resonance	Pixelated array (100 pixels)	—	Protein (Sim.)
Niu et al. [56]	THz (1.5–2.5 THz)	Au (with 50 nm Ti)	EIT	Pixelated array (10 pixels)	LOD: 1.76 [μg/mm^2^]	L‐tyrosine (Exp.)
This work	THz (0.3–0.8 THz)	Al (adhesion‐free, 200 nm)	BZF‐BIC	Pixelated array (49 pixels)	EF: 14×	*cis*‐AB (Exp.)

## 4 Conclusion

In conclusion, we have proposed and experimentally demonstrated a broadband, high‐sensitivity THz sensing platform leveraging the scalable high‐*Q* properties of perturbed BZF‐BIC in a plasmonic metasurface. The radiating and topological properties of the designed BZF‐BIC mode were systematically analyzed. By introducing structural perturbations, we successfully transformed this *C*_2_ symmetry‐protected nonradiative state into a radiative q‐BIC mode, achieving high‐*Q* resonances with significant near‐field enhancement. Furthermore, the periodic lattice perturbation of BZF‐BICs endows the resonances with robustness against fabrication flaws during the fine dimensional scaling process. Based on this mechanism, a distributed array of resonant probes was developed across multiple frequency channels, enabling the high‐sensitivity retrieval of molecular absorption fingerprints over a broad THz range. The metasensor's performance was numerically verified using three representative analytes, *α*‐lactose, *cis*‐AB, and benzoic acid, within the 0.3–0.8 THz range. Experimental validation using *cis*‐AB further confirmed its capability for both molecular fingerprint retrieval and quantitative concentration detection. Additionally, the design can be easily extended to other spectral regions by simply scaling the geometric parameters, offering a versatile solution for chemical and biological sensing. These results demonstrate the significant potential of the proposed metasensor for practical THz applications, such as label‐free diagnostics and on‐chip spectroscopic systems.

## Author Contributions

**Yihan Xu:** conceptualization, methodology, software, validation, writing – original draft. **Xieyu Chen:** supervision, writing – review and editing. **Shoujun Zhang:** investigation. **Lu Zhou:** resources. **Zhen Tian:** conceptualization, supervision, validation, resources, project administration, writing – review and editing.

## Conflicts of Interest

The authors declare no conflicts of interest.

## Supporting information


Supporting Information S1

## Data Availability

The data that support the findings of this study are available from the corresponding author upon reasonable request.
