# Supplementary material for: Broadband THz Molecular Fingerprinting Enabled by Brillouin Zone Folded Quasi‐BICs in Plasmonic Metasurfaces
Source: Nanophotonics. 2026 Apr 16;15(8):e70076. doi: 10.1002/nap2.70076 (PMC13642549; doi:10.1002/nap2.70076)
Supplement: Supplementary file 1 [file NAP2-15-e70076-s001.docx]

**Supporting Information:**

**Broadband THz Molecular Fingerprinting Enabled by Brillouin Zone Folded Quasi-BICs in Plasmonic Metasurfaces**

*Yihan Xu*^1^, *Xieyu Chen*^*1^, *Shoujun Zhang*^1^, *Lu Zhou*^2^, and *Zhen Tian*^*1^

^1^Center for Terahertz Waves and College of Precision Instrument and Optoelectronics Engineering, State Key Laboratory of Precision Measuring Technology and Instruments,

Tianjin University, Tianjin 300072, China

E-mail: chenxieyu@tju.edu.cn (*Xieyu Chen*); tianzhen@tju.edu.cn (*Zhen Tian***)**

^2^School of Chemistry and Pharmaceutical Engineering, Shandong First Medical University & Shandong Academy of Medical Sciences, Jinan 250021, China

Contents

S1. Material Selection and the Impact of Native Oxide Layer	1

S1.1. Rationale for Choosing Aluminum	1

S1.2. Impact of the Native Aluminum Oxide Layer	1

S2. Fabrication Flowchart of the Metasensor	3

S3. Reusability of the Metasensor and Cleaning Protocol	4

S4. Envelope Extraction Algorithm and Reconstruction Error	5

# S1. Material Selection and the Impact of Native Oxide Layer

## S1.1. Rationale for Choosing Aluminum

To evaluate the optical performance of different plasmonic materials, we simulated the metasurface using aluminum (Al), gold (Au), and silver (Ag) with their default material properties from the CST library. As shown in Figure S1a, the transmission spectra of the three metals are nearly indistinguishable, indicating that Al provides comparable performance to noble metals. Given this optical equivalence, Al was selected as the constituent material over noble metals or all-dielectric ceramics for several practical reasons:

Cost-effectiveness and Process Simplicity: While gold and silver are excellent plasmonic materials, they exhibit poor adhesion to silicon substrates and typically require an additional adhesion layer (such as Ti or Cr)^[1, 2]^. Aluminum adheres strongly to silicon natively, eliminating the need for this extra deposition step and avoiding any potential ohmic losses introduced by the adhesion layer. Furthermore, aluminum is highly cost-effective.

Fabrication Simplicity: Compared to all-dielectric ceramic resonators (e.g., TiO_2_ or ZrO_2_),^[3]^ which are notoriously difficult to pattern due to their chemical inertness and hardness—often requiring complex dry etching (e.g., ion beam milling) or bottom-up self-assembly processes—aluminum allows for rapid, large-area, and low-cost fabrication using standard photolithography and simple wet etching.

## S1.2. Impact of the Native Aluminum Oxide Layer

In ambient environments, a thin native oxide layer (Al_2_O_3_, typically 2–4 nm thick) naturally forms on the surface of aluminum microstructures. To investigate its potential impact on the high-*Q* resonance properties, we performed comparative full-wave simulations.

In the simulation, the Al_2_O_3_ layer was modeled using the built-in Alumina (loss free) material from the CST material library. As shown in Figure S1b, the transmission spectrum of the oxidized aluminum resonators nearly perfectly overlaps with that of the pure aluminum resonators. Specifically, the introduction of the 4-nm oxide layer causes a minuscule frequency shift (< 1 GHz) and a negligible amplitude variation of ~ 0.1%. Such a sub-GHz shift is practically unobservable in experiments. This is fundamentally because the operating wavelength in the THz regime (~ 300-100 μm) is approximately five orders of magnitude larger than the thickness of the oxide layer. Consequently, the deeply subwavelength native oxide layer introduces virtually zero near-field perturbation, confirming that pure aluminum is a reliable material choice for THz metasensors.

**Figure S1**. (a) Simulated transmission spectra of the metasurface unit cell using Al, Au, and Ag resonators. (b) Simulated transmission spectra of pure Al resonators (black line) and Al resonators conformally coated with a 4-nm-thick Al_2_O_3_ layer (red line). The right panels illustrate the corresponding 3D geometric models.

# S2. Fabrication Flowchart of the Metasensor

The metasurface sensors were fabricated on high-resistivity silicon substrates using standard ultraviolet (UV) photolithography combined with wet etching techniques, as illustrated in Figure S2. First, a 2-mm-thick high-resistivity silicon wafer was ultrasonically cleaned with acetone, absolute ethanol, and deionized water, and then dried with nitrogen gas. Subsequently, a 200-nm-thick aluminum film was deposited onto the cleaned substrate via thermal evaporation. Then, a positive photoresist was spin-coated onto the aluminum layer and pre-baked on a hotplate. After precise alignment with a pre-designed photomask, the sample was exposed to UV light and immersed in a developer solution to transfer the metasurface patterns into the photoresist. Next, the exposed aluminum was selectively removed using a standard wet etchant mixture (phosphoric, acetic, and nitric acids). Finally, the remaining photoresist was stripped using acetone, followed by thorough rinsing and drying, yielding the final metasurface sensor arrays.

**Figure S2**. Schematic illustration of the step-by-step fabrication process of the metasurface sensor.

# S3. Reusability of the Metasensor and Cleaning Protocol

A significant advantage of the proposed aluminum-based metasensor is its reusability. The sensor can be regenerated after each measurement by removing the analyte layer using standard solvent washing. For instance, *cis*-AB can be thoroughly dissolved by rinsing the chip with acetone, followed by absolute ethanol and deionized water, and finally blowing it dry with high-purity nitrogen gas. For water-soluble analytes like *α*-lactose, deionized water is used, whereas absolute ethanol is utilized for benzoic acid. In our experiments, multiple tests were consecutively performed on the exact same metasurface chip. When cleaned promptly after a measurement, the robustness of the aluminum resonators ensures that the baseline transmission spectrum is fully recovered without any degradation in the high-*Q* resonance performance. This excellent reusability significantly reduces the fabrication cost and enables the continuous detection of various analytes using a single metasensor chip.

# S4. Envelope Extraction Algorithm and Reconstruction Error

To extract the continuous absorption envelope from the discrete metasurface arrays, we employed a straightforward and computationally robust method. Specifically, the baseline transmission dip values of the bare metasurface (*t*_0_) were subtracted from those of the analyte-coated metasurface (*t*_s_) at each of the 49 discrete frequency points (spaced by 10 GHz). A standard cubic spline interpolation was then applied to these 49 discrete positive signal points (*t*_s_ - *t*_0_) to reconstruct the continuous envelope.

To quantitatively evaluate the accuracy of this extraction algorithm, we calculated the reconstruction error between our metasurface-retrieved envelope and the standard reference absorption spectrum of bulk *cis*-AB measured via conventional THz-TDS. Because the metasurface transmission difference and the bulk absorption coefficient have different physical units, both spectra were normalized to compare their lineshapes. As shown in Figure S3a, the simulated envelope reconstruction exhibits a near-perfect match with the reference spectrum, yielding a Pearson correlation coefficient of 0.994 and a Normalized Root Mean Square Error (NRMSE) of 9.52%. This confirms that the 10-GHz discrete sampling interval is theoretically dense enough to capture the broad absorption features accurately. For the experimental demonstration (Figure S3b, corresponding to the 25 mg/mL concentration in Figure 4b), despite the presence of practical system noise and baseline fluctuations, the reconstructed envelope still maintains high fidelity, achieving a Pearson correlation coefficient of 0.939 and an NRMSE of 10.72%. These quantitative metrics conclusively demonstrate the reliability of the proposed discrete sampling and spline interpolation method for broadband fingerprint retrieval.

Figure S3. Quantitative evaluation of the envelope reconstruction fidelity for (a) the simulated data and (b) the experimental data (25 mg/mL *cis*-AB). The black solid lines represent the normalized reference absorption spectrum, the red dots are the 49 discrete signals extracted from the metasensor, and the red dashed lines are the reconstructed envelopes using cubic spline interpolation.

**References**

[S1]	MADSEN S J, ESFANDYARPOUR M, BRONGERSMA M L, et al. Observing Plasmon Damping Due to Adhesion Layers in Gold Nanostructures Using Electron Energy Loss Spectroscopy [J]. ACS Photonics, 2017, 4(2): 268-274.

[S2]	HABTEYES T G, DHUEY S, WOOD E, et al. Metallic Adhesion Layer Induced Plasmon Damping and Molecular Linker as a Nondamping Alternative [J]. ACS Nano, 2012, 6(6): 5702-5709.

[S3]	CHEN X, XIN S, LIU Q, et al. Dielectric metasurface-assisted terahertz sensing: mechanism, fabrication, and multiscenario applications [J]. Nanophotonics, 2025, 14(3): 271-296.
